# Charcot‐Leyden crystals in tissues

**DOI:** 10.1002/clt2.12275

**Published:** 2023-07-01

**Authors:** Tomoko Sasaki, Isao Suzaki, Shigeharu Ueki

**Affiliations:** ^1^ Department of General Internal Medicine and Clinical Laboratory Medicine Akita University Graduate School of Medicine Akita Japan; ^2^ Department of Otorhinolaryngology Head and Neck Surgery School of Medicine Showa University Tokyo Japan

**Keywords:** ENT, eosinophil, immunology, rhinosinusitis

To the Editor,

We read with interest the paper by Chen et al. entitled “Predictive significance of Charcot‐Leyden crystal (CLC) structures for nasal polyp recurrence”.^1^ CLCs are formed when galectin‐10 crystallizes intracellularly or extracellularly through eosinophil ETosis, an active cell death process involving the disruption of the cell nucleus and plasma membrane to release web‐like chromatin structures.^2^, ^3^ Intact eosinophil granules are also released during this process, typically characterized by the presence of cell‐free eosinophil granules (Cfegs).^2^ In the H&E‐stained specimens shown by Chen et al., most eosinophils in the nasal polyp tissues have barely retained their morphology, and both cell disruption with web‐like nuclear contents and Cfegs are clearly visible. Meanwhile, the lack of concordance between eosinophil counts and CLCs in the tissues may have arisen because cytolytic eosinophils are not evaluated.^4^ Not only the cytotoxic granule proteins released by ETosis but also the presence of the crystals themselves may enhance the exacerbation loop of inflammation.^5^ Therefore, it is significant to focus on the presence of CLCs in the tissues.

We previously examined the nasal polyp histology in patients with eosinophilic chronic rhinosinusitis and found that CLCs were present in more than half of the patients with moderate to severe disease based on the JESREC criteria.^6^ In the JESREC study, the recurrence rates were 31.1% in patients with moderate disease and 51.8% in patients with severe disease.^6^ These findings are generally consistent with the results of Chen et al. Nasal polyps are clinically easy to obtain for examination, and pathologists should be encouraged to report the presence of CLCs to clinicians. However, there are several associated problems. First, the tissue sections are not uniform and the results can differ markedly depending on the field observed. Second, depending on the angle at which the crystals are sectioned, the characteristic needle‐like structures may be difficult to identify. Third, CLCs are difficult to distinguish from other eosinophilic structures, including collagen fibers. As shown in Figure 1, CLCs can be easily observed by immunofluorescence staining for galectin‐10,^3^ but this is a time‐consuming and labor‐intensive process. Charcot‐Leyden crystals have also been observed in tissues from patients with other eosinophilic diseases, but their clinical significance has not been fully investigated. It will be interesting to determine whether similar trends are present in other diseases and whether CLCs can predict the efficacy of eosinophil‐targeted therapies.

**FIGURE 1:**
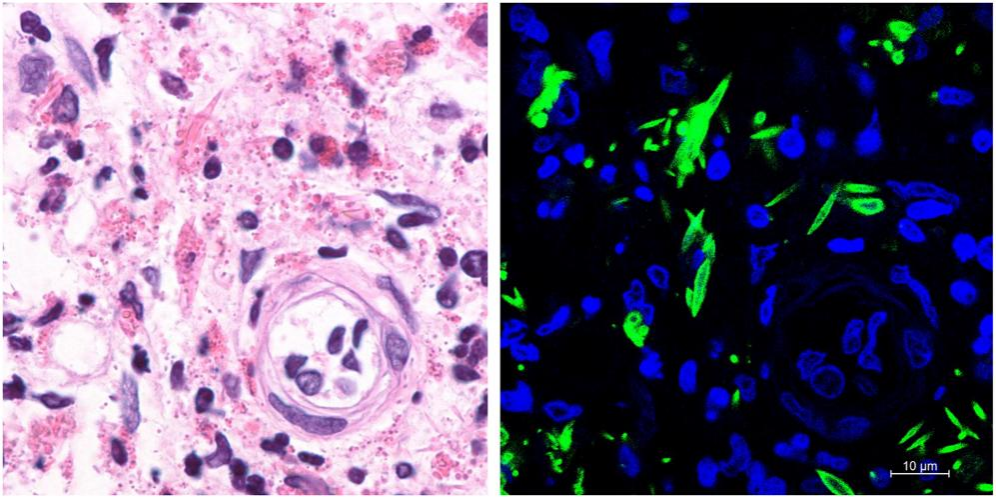
The same nasal polyp section from a patient with eosinophilic chronic rhinosinusitis was evaluated by (left) H&E staining and (right) immunofluorescence staining for galectin‐10 (green) and DNA (blue). Abundant Charcot‐Leyden crystals (CLCs) were clearly observed as galectin‐10‐positive structures. Written informed consent was obtained from a patient in accordance with the principles laid out in the Declaration of Helsinki and using Institutional Review Board‐approved protocols.

## Author Contributions

**Tomoko Sasaki**: Investigation (Equal); Validation (Equal); Visualization (Equal). **Isao Suzaki**: Conceptualization (Equal); Investigation (Equal); Resources (Supporting).

## CONFLICT OF INTEREST STATEMENT

Shigeharu Ueki received grants and honoraria for lectures from AstraZeneca, honoraria for lectures from GlaxoSmithKline and Sanofi, and grants from Novartis, VIB, and Maruho Co. Ltd.

## FUNDING INFORMATION

Japan Society for the Promotion of Science, Grant/Award Numbers: 20H03832, 20K08794, 21K07833, 21K08434

## Data Availability

Data available on request from the authors.
